# Association of platelet to HDL-C ratio with short-term mortality in critically ill intracerebral hemorrhage patients: a MIMIC-IV analysis

**DOI:** 10.1038/s41598-026-43526-4

**Published:** 2026-03-10

**Authors:** Yongtong He, Qianshan Zhao, Qiyin Cai

**Affiliations:** 1 Department of Neurosurgery, Affiliated Jiangmen Traditional Chinese Medicine (TCM) Hospital of Ji’nan University, No.30, Huayuan East Road, Jiangmen, 529000 Guangdong China; 2 Department of Pediatric Intensive Care Units, Jiangmen Maternity and Child Health Care Hospital, Jiangmen, China

**Keywords:** Platelet to high-density lipoprotein cholesterol ratio (PHR), Intracerebral hemorrhage (ICH), Short-term mortality, MIMIC-IV, Biomarkers, Diseases, Medical research, Risk factors

## Abstract

Intracerebral hemorrhage (ICH) is associated with substantial early mortality, yet prognostic biomarkers integrating coagulation, inflammation, and lipid metabolism are limited. The platelet to high-density lipoprotein cholesterol ratio (PHR) may reflect this balance. In a retrospective cohort of 878 critically ill ICH patients from the MIMIC-IV database (mean age 69.6 ± 13.8 years, 55.0% female), overall in-hospital and 30-day mortality were 15.0% and 20.5%, respectively. Short-term mortality was defined as death occurring either during hospitalization (in-hospital mortality) or within 30 days of admission (30-day mortality). Higher PHR at ICU admission was independently associated with lower short-term mortality. Each 1-SD increase corresponded to a 20–28% reduction in risk for in-hospital (adjusted HR 0.72, 95% CI 0.59–0.87) and 30-day mortality (HR 0.80, 95% CI 0.68–0.94). When analyzed by quartiles, patients in the highest PHR group had the lowest mortality (in-hospital HR 0.49, 95% CI 0.29–0.83; 30-day HR 0.55, 95% CI 0.35–0.88), consistent with Kaplan-Meier survival analyses. Restricted cubic spline analysis indicated a linear inverse relationship. Results were robust in subgroup analyses and largely consistent in sensitivity analyses, with modest attenuation for in-hospital mortality. These findings suggest PHR has an independent inverse relationship with short-term mortality. Important prognostic factors, including hematoma volume and location, were unavailable; PHR may reflect underlying disease severity rather than causal protection.

**Supplementary Information:**

The online version contains supplementary material available at 10.1038/s41598-026-43526-4.

## Introduction

Intracerebral hemorrhage (ICH) remains one of the most devastating forms of stroke, contributing disproportionately to global morbidity, mortality, and healthcare burden^1^. Compared with ischemic stroke, ICH is associated with markedly higher early mortality and poorer functional outcomes. The global incidence of ICH is estimated at approximately 29.9 per 100,000 person-years, with a greater prevalence in the elderly population^2^. Early hematoma expansion, perihematomal edema, and subsequent inflammatory and coagulopathic cascades are major determinants of acute neurological deterioration and death, highlighting the urgent need for prognostic biomarkers that integrate coagulation, inflammation, and lipid metabolism^3,4^. The platelet-to-high-density lipoprotein cholesterol ratio (PHR) is a composite index that may capture a critical balance within this pathophysiology. Platelets are central to coagulation and contribute to the inflammatory cascade, whereas HDL-C exerts anti-inflammatory, antioxidant, and endothelial-protective effects, and and may modulate platelet function and coagulation.Thus, PHR reflects the equilibrium between pro-thrombotic/pro-inflammatory forces and vascular-protective/anti-inflammatory mechanisms—a balance hypothesized to be closely associated with early outcomes after ICH^5–7^.

Individual circulating biomarkers offer limited prognostic information in ICH, as each reflects only a single pathophysiological axis. Platelets are actively involved in neuroinflammation and coagulopathy, thereby promoting perihematomal edema and microvascular thrombosis^8^. In contrast, high-density lipoprotein cholesterol (HDL-C) exerts anti-inflammatory and antioxidant effects and supports endothelial function, with higher levels being associated with favorable outcomes in several cohorts^9,10^. The PHR integrates into a single composite index of thrombotic risk versus lipid-mediated protection. By capturing this balance, PHR has demonstrated superior predictive value over its individual components in inflammatory and metabolic disorders, such as periodontitis^11^ and cardiovascular disease^12,13^.

However, the relationship between PHR and short-term mortality in ICH, especially in critically ill patients, has not been well characterized. This study therefore aims to assess the association between PHR and short-term mortality in critically ill ICH patients.

## Results

### Baseline characteristics of patients

**Fig. 1:**
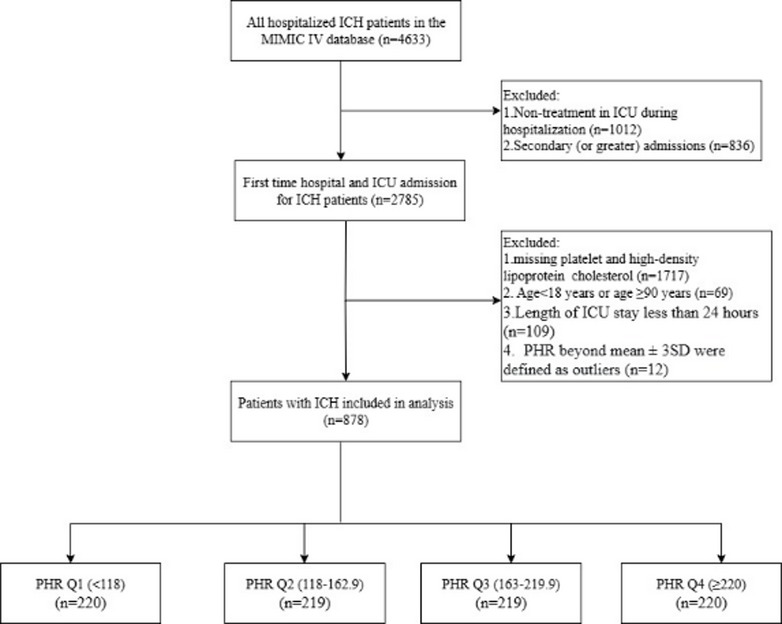
Flowchart of participant selection.

A total of 4,633 hospitalized patients with ICH were initially identified from the MIMIC-IV database. After sequential exclusions based on eligibility criteria, 878 patients with complete data were retained for the final analysis (Fig. 1).

Table 1 presents baseline characteristics according to PHR quartiles (Q1: <118; Q2: 118–162.9; Q3: 163–219.9; Q4: ≥220). The mean PHR was 176.5 ± 81.6, with a median of 163.1 (interquartile range: 117.7–219.3). The cohort had a mean age of 69.6 ± 13.8 years, with 55.0% being female. Significant differences across quartiles were observed in age, gender, weight, MAP, diabetes, hemoglobin, WBC, glucose, eGFR, and SAPSII (all *P* < 0.05). The overall in-hospital mortality was 15.0%, with group-specific rates of 17.3%, 17.8%, 12.3%, and 12.7%. The 30-day mortality was 20.5%, corresponding to group rates of 26.8%, 19.2%, 21.0%, and 15.0%.

**Table 1:** Baseline clinical characteristics of the study population.

Variables	PHR	Q1 (< 118)	Q2 (118-162.9)	Q3 (163-219.9)	Q4 (≥ 220)	*P* value
	Total (*n* = 878)	*n* = 220	*n* = 219	*n* = 2219	*n* = 220	
General characteristics						
Age, (years)	69.6 ± 13.8	73.3 ± 12.7	71.3 ± 12.7	69.2 ± 14.1	64.7 ± 14.2	< 0.001
gender, (female), n (%)	483 (55.0)	106 (48.2)	133 (60.7)	116 (53)	128 (58.2)	0.04
Race, (white), n (%)	481 (54.8)	119 (54.1)	131 (59.8)	125 (57.1)	106 (48.2)	0.085
Weight, (kg)	80.6 ± 22.6	73.0 ± 18.1	79.5 ± 19.8	82.1 ± 24.1	87.7 ± 25.2	< 0.001
Vital signs						
Heart rate, (beats/min)	79.7 ± 13.5	78.8 ± 12.6	79.3 ± 13.6	80.0 ± 13.6	80.9 ± 14.1	0.392
Respiratory rate, (breaths/min)	18.8 ± 2.9	18.6 ± 2.9	18.6 ± 2.6	18.7 ± 3.0	19.2 ± 2.8	0.064
MAP, (mmHg)	88.1 ± 10.2	86.3 ± 10.3	88.8 ± 9.5	88.3 ± 10.0	88.8 ± 11.0	0.023
Commodities						
Hypertension, n (%)	573 (65.3)	138 (62.7)	148 (67.6)	147 (67.1)	140 (63.6)	0.629
Diabetes, n (%)	263 (30.0)	40 (18.2)	73 (33.3)	67 (30.6)	83 (37.7)	< 0.001
Laboratory parameters						
Hemoglobin, (g/dl)	12.7 ± 1.9	12.4 ± 1.7	12.9 ± 1.8	12.7 ± 1.8	12.7 ± 2.1	0.028
WBC, (10^9/L)	10.0 (8.0, 12.8)	8.7 (6.9, 11.1)	9.5 (7.6, 11.9)	10.8 (8.8, 13.7)	10.8 (8.6, 14.1)	< 0.001
Glucose, (mg/dl)	136.1 ± 43.3	127.2 ± 34.8	136.6 ± 44.1	138.0 ± 44.2	142.5 ± 47.9	0.002
PT, (s)	13.1 ± 3.6	13.4 ± 3.6	13.1 ± 4.1	13.0 ± 3.8	13.0 ± 2.5	0.722
APTT, (s)	29.2 ± 7.9	28.9 ± 5.1	28.8 ± 7.5	29.9 ± 10.7	29.0 ± 7.3	0.405
eGFR, (mL/min/1.73 m^2^)	88.4 ± 37.8	93.1 ± 43.5	86.3 ± 34.2	90.7 ± 36.4	83.4 ± 36.0	0.032
Scoring system						
SAPSII, (scores)	31.9 ± 10.6	33.9 ± 10.8	31.9 ± 9.2	31.2 ± 10.5	30.6 ± 11.5	0.007
ASPIII, (scores)	40.9 ± 18.2	41.6 ± 17.3	39.9 ± 18.4	40.1 ± 16.4	41.8 ± 20.4	0.593
GCS, (scores)	11.1 ± 3.4	10.9 ± 3.4	11.2 ± 3.2	11.0 ± 3.3	11.2 ± 3.6	0.785
Outcomes						
In-hospital mortality	132 (15.0)	38 (17.3)	39 (17.8)	27 (12.3)	28 (12.7)	0.226
30-day mortality	180 (20.5)	59 (26.8)	42 (19.2)	46 (21)	33 (15)	0.021

Note: PHR: platelet to high-density lipoprotein cholesterol ratio; Q: quartile; MAP: mean arterial pressure; WBC: white blood cell count; PT, prothrombin time; APTT: activated partial thromboplastin time; eGFR: estimated glomerular filtration rate; SAPSII: simplified acute physiology score II; ASPIII: acute physiology score III; GCS: glasgow coma scale.

Compared to excluded patients, the final analysis cohort had lower disease severity and mortality rates (Table S6). Among excluded patients, those with ICU stays < 24 h had the highest mortality (Table S7), indicating that outcomes in this subgroup were strongly influenced by extreme early clinical severity and heterogeneous early trajectories.

### PHR and in-hospital and 30-day mortality

Multivariable Cox regression was used to evaluate the association between PHR and both in-hospital and 30-day mortality (Table 2).

**Table 2:** Association between PHR and short-term mortality in the multiple cox regression model.

Variable	Total(*n*)	Model 1	*P* value	Model 2	*P* value
		HR (95% CI)		HR (95% CI)	
Mortality of in-hospital					
Pre 1 SD increase	878	0.82 (0.68–0.98)	0.032	0.72 (0.59–0.87)	0.001
Quartiles of PHR					
Q1	220	1(Ref)		1(Ref)	
Q2	219	1.04 (0.66–1.62)	0.879	0.99 (0.62–1.59)	0.976
Q3	219	0.67 (0.41–1.09)	0.108	0.59 (0.35–0.99)	0.045
Q4	220	0.66 (0.41–1.08)	0.098	0.49 (0.29–0.83)	0.008
*P* value for trend test			0.034		0.002
Mortality of 30-day					
Pre 1 SD increase	878	0.79 (0.67–0.93)	0.005	0.8 0(0.68–0.94)	0.005
Quartiles of PHR					
Q1	220	1(Ref)		1(Ref)	
Q2	219	0.71 (0.48–1.05)	0.089	0.95 (0.62–1.44)	0.801
Q3	219	0.76 (0.51–1.11)	0.155	0.89 (0.59–1.35)	0.590
Q4	220	0.51 (0.34–0.79)	0.002	0.55 (0.35–0.88)	0.012
*P* value for trend test			0.004		0.017

Model 1: Unadjusted.

Model 2: adjusted for age, gender, heart rate, respiratory rate, MAP, hypertension, diabetes, WBC, glucose, APTT, eGFR, SAPSII and ASPIII.

HR: hazard ratio; CI: confidence interval; Ref: reference; PHR: platelet to high-density lipoprotein cholesterol ratio; Q: quartile; MAP: mean arterial pressure; WBC: white blood cell count; APTT: activated partial thromboplastin time; eGFR: estimated glomerular filtration rate; SAPSII: simplified acute physiology score II; ASPIII: acute physiology score III.

For in-hospital mortality, crude analysis (Model 1) showed that each 1-SD increase in PHR was associated with an 18% lower risk (HR = 0.82, 95% CI 0.68–0.98, *P* = 0.032). After full adjustment for age, gender, heart rate, respiratory rate, MAP, hypertension, diabetes, WBC, glucose, APTT, eGFR, SAPS II, and APS Ⅲ (Model 2), the association remained significant (HR = 0.72 per 1-SD increase, 95% CI 0.59–0.87, *P* = 0.001). When PHR was analysed in quartiles, adjusted HRs relative to Q1 were 0.99 (95% CI 0.62–1.59), 0.59 (95% CI 0.35–0.99), and 0.49 (95% CI 0.29–0.83) for Q2, Q3, and Q4, respectively (*P* for trend = 0.002).

For 30-day mortality, each 1-SD increase in PHR was similarly associated with reduced risk in both Model 1 (HR = 0.79, 95% CI 0.67–0.93, *P* = 0.005) and Model 2 (HR = 0.80, 95% CI 0.68–0.94, *P* = 0.005). In the quartile analysis (Model 2), adjusted HRs compared with Q1 were 0.95 (95% CI 0.62–1.44), 0.89 (95% CI 0.59–1.35), and 0.55 (95% CI 0.35–0.88) for Q2, Q3, and Q4, respectively (*P* for trend = 0.017).

### The linear association between PHR and in-hospital and 30-day mortality

**Fig. 2:**
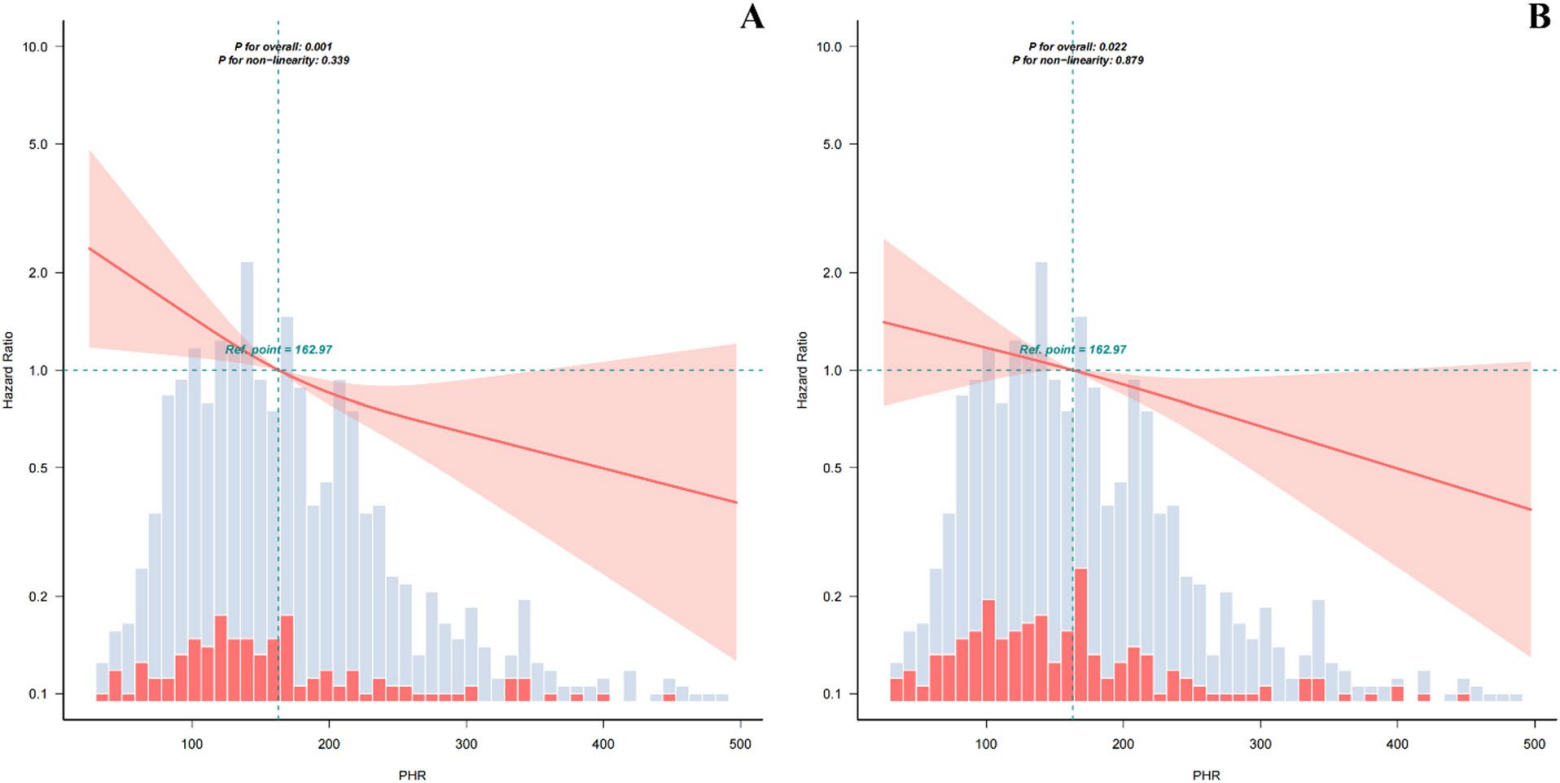
Liner dose-response relationship between PHR and short-term mortality of ICU patients with ICH. Panel A: mortality of in-hospital, Panel B: mortality of 30-day. Solid deep red lines are multivariable-adjusted HRs. Light red areas are the 95% confidence intervals derived from restricted cubic spline regressions with 4 knots. Dashed black lines are reference lines for no association at a hazard ratio of 1.0. Mortality of in-hospital and mortality of 30-day decrease as PHR increased. Cox model adjustments: age, gender, heart rate, respiratory rate, MAP, hypertension, diabetes, WBC, glucose, APTT, eGFR, SAPS II, APS Ⅲ. The flattening of the curve beyond PHR = 230 in Panel A may reflect reduced precision due to sparse data (*n* < 200) and is consistent with the results of the exploratory piecewise analysis. PHR: platelet to high-density lipoprotein cholesterol ratio; ICU: intensive care unit; ICH: Intracerebral hemorrhage; MAP: mean arterial pressure; WBC: white blood cell count; APTT: activated partial thromboplastin time; eGFR: estimated glomerular filtration rate; SAPSII: simplified acute physiology score II; ASPIII: acute physiology score Ⅲ.

Figure 2A-B demonstrates a linear inverse relationship between PHR and both mortality outcomes. Tests for overall association were significant (*P* = 0.001 for in-hospital mortality; *P* = 0.022 for 30-day mortality), whereas tests for non-linearity were not significant (*P* = 0.339 and *P* = 0.879, respectively), consistent with the quartile-based HRs presented in Table 2. Exploratory two-piecewise Cox regression identified a potential inflection point at PHR = 230 (likelihood ratio test *P* = 0.028, Table S8), with PHR showing a significant inverse association with mortality below this threshold (HR 0.994, 95% CI 0.990–0.998, *P* = 0.006) but not above it (HR 0.997, 95% CI 0.988–1.007, *P* = 0.589).

### Kaplan-meier survival curve analysis

**Fig. 3:**
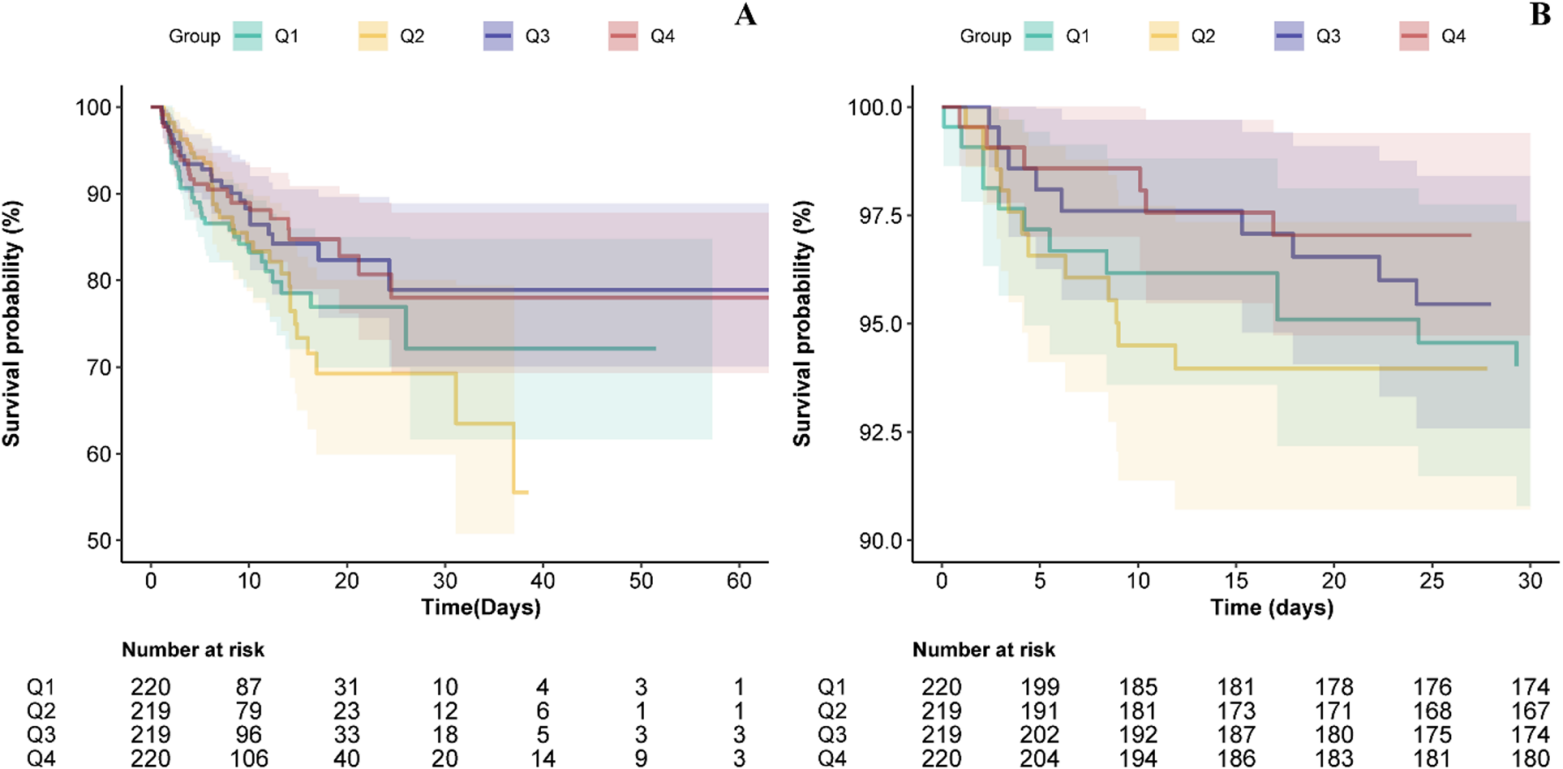
Kaplan-Meier survival analysis of short-term mortality with PHR in four groups. Panel A: mortality of in-hospital, Panel B: mortality of 30-day. Q1-Q4 represent quartiles from lowest to highest PHR (Q1: *n* = 220; Q2: *n* = 220; Q3: *n* = 220; Q4: *n* = 220). Shaded areas around each survival curve indicate the 95% confidence intervals. Log-rank test demonstrated significant differences in survival probability across quartiles (Panel A: *P* < 0.05; Panel B: *P* < 0.05). For in-hospital mortality, survival days were displayed up to 60 days.

Figure 3A-B presents Kaplan-Meier survival curves for in-hospital and 30-day mortality by PHR quartiles. Patients in Q3–Q4 demonstrated significantly higher in-hospital survival compared with those in Q1–Q2 (log-rank *P* < 0.05). For 30-day mortality, the highest survival probability was observed in Q4 (log-rank *P* < 0.05).

### Subgroup analysis

**Fig. 4:**
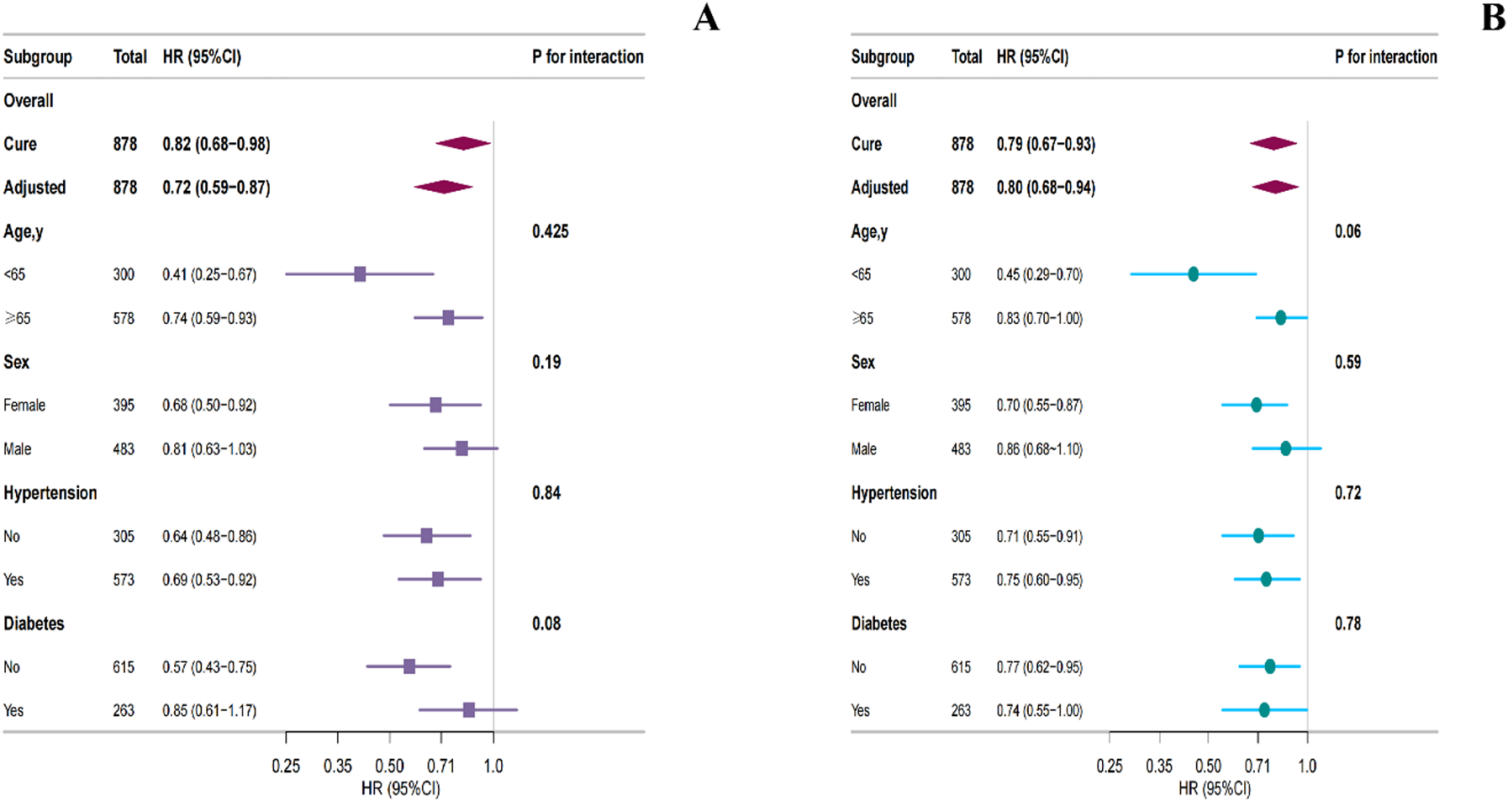
Forest plots of the association between PHR and short-term mortality in different subgroups. Panel A: mortality of in-hospital, Panel B: mortality of 30-day. The two panels present multivariable-adjusted hazard ratios (HRs) and 95% confidence intervals (CIs) for the association between PHR and (**A**) mortality of in-hospital and (**B**) mortality of 30-day across prespecified subgroups, estimated with Cox proportional-hazards models. Adjustments were made for age, gender, heart rate, respiratory rate, mean arterial pressure, hypertension, diabetes, white-blood-cell count, glucose, APTT, eGFR, SAPS II and APS Ⅲ. Subgroup analyses were stratified by age, gender, hypertension and diabetes; P values for interaction test the null hypothesis of no effect modification by the subgrouping variable. In Panel A, higher PHR remained significantly associated with in-hospital mortality in every subgroup, with no evidence of significant interaction (all *P* interaction > 0.05). In Panel B, the association between PHR and 30-day mortality was consistently significant in most subgroups. However, a trend toward significant effect modification was noted for age (*P* for interaction = 0.06),​ with a more pronounced protective association in patients aged < 65 years (adjusted HR = 0.45, 95% CI: 0.29–0.70) compared to those aged ≥ 65 years (adjusted HR = 0.83, 95% CI: 0.70–1.00). This finding suggests potential heterogeneity in the strength of the association by age, though it did not reach the conventional threshold for statistical significance (*P* < 0.05). PHR: platelet to high-density lipoprotein cholesterol ratio; MAP: mean arterial pressure; WBC: white blood cell count; APTT: activated partial thromboplastin time; eGFR: estimated glomerular filtration rate; SAPSII: simplified acute physiology score II; ASPIII: acute physiology score Ⅲ.

Subgroup analyses of the association between PHR (per 1-SD increase) and in-hospital and 30-day mortality are shown in Fig. 4A-B. Multivariable adjustment revealed broadly consistent HR across most prespecified subgroups, including gender, hypertension, and diabetes strata (all *P* for interaction > 0.05). For 30-day mortality, a trend toward effect modification was observed for age (*P* for interaction = 0.06), with a more pronounced inverse association in patients aged < 65 years.

### Sensitivity analysis

In sensitivity analyses including patients with ICU stays < 24 h and statistical outliers, the inverse association between PHR (per 1-SD) and in-hospital mortality was attenuated but remained directionally consistent, with significant protection in the fully adjusted model (HR = 0.77, 95% CI 0.63–0.94, *P* = 0.01; Q4 vs. Q1: HR = 0.53, 95% CI 0.32–0.88, *P* = 0.014). The corresponding trend analysis showed that each quartile increase in PHR was associated with a lower risk of in-hospital mortality (HR for trend = 0.79, 95% CI 0.68–0.96, *P* = 0.00). The inverse association with 30-day mortality remained stable and statistically significant (HR = 0.79, 95% CI 0.66–0.95, *P* = 0.010), consistent with the primary analysis. Similarly, each quartile increase in PHR was associated with reduced 30-day mortality (HR for trend = 0.85, 95% CI 0.74–0.97, *P* = 0.015) (Table S9).

## Discussion

In this retrospective cohort of critically ill patients with ICH from the MIMIC‑IV database, we observed a robust, independent inverse association between PHR and short‑term mortality, which we defined as death occurring either during hospitalization (in-hospital mortality) or within 30 days of admission (30-day mortality). Each 1‑SD increase in PHR was associated with a 20–28% lower risk of in‑hospital and 30‑day mortality, with the highest PHR quartile showing the lowest mortality. These associations were consistent across prespecified demographic and clinical subgroups and remained directionally consistent in sensitivity analyses, with modest attenuation for in-hospital mortality. Notably, in an exploratory analysis, the inverse association with 30-day mortality appeared more pronounced in patients younger than 65 years (*P* for interaction = 0.06), though this requres validation. Importantly, these findings reflect associations only and do not imply causality.

In contrast to the elevated PHR levels typically associated with pro-thrombotic and pro-inflammatory risk in arterial occlusive and metabolic disorders^14,15^, our ICH cohort demonstrates an inverse association: higher admission PHR predicts lower short-term mortality, with the highest quartile exhibiting the lowest in-hospital and 30-day death risk. This divergence may reflect the unique pathophysiology of acute intracerebral hemorrhage. Rather than representing intrinsic hemostatic reserve, a higher platelet count at admission may reflect greater platelet availability in the acute systemic milieu. PHR may therefore serve as an integrative indicator of physiological reserve. Its association with reduced hematoma expansion risk is likely mediated through this broader resilient state, not a direct hemostatic effect^16^. Furthermore, this association aligns with preclinical evidence suggesting that HDL-C may confer vascular-protective effects, including mitigation of perihematomal edema in experimental ICH models^17^. In contrast, elevated PHR in cardiovascular and metabolic populations typically indicates higher risk via enhanced platelet activation and impaired HDL antioxidant function. In ICH, PHR likely reflects acute vascular rupture tolerance—a balance between platelet-mediated hemostasis and HDL-mediated microvascular protection^15,18,19^. However, these mechanistic interpretations remain speculative and hypothesis-generating; PHR should be interpreted as a marker of physiological state at admission rather than a mediator of protection.

Our observations are not without precedent in acute brain injury literature. Higher platelet counts in traumatic brain injury^20^ and higher platelet-to-neutrophil ratios in acute ischemic stroke^21^ have been associated with better outcomes, supporting the concept that preserved platelet availability and favorable platelet-inflammatory balance may be context-dependent in acute brain injury. This provides a broader framework for interpreting our findings in ICH.

Several hypothese may explain the observed inverse association between PHR and short‑term mortality in ICH patients. It is possible that higher PHR reflects greater platelet availability, which may support hemostatic clot formation and potentially limit hematoma expansion, a known determinant of early death. Platelets may also play a role in maintaining vascular integrity and modulating inflammatory responses^22,23^. HDL-C has been suggested in preclinical studies to exerts anti-inflammatory, antioxidant, and endothelial-protective effects that could mitigateperihematomal injury^24,25^. However, these mechanistic interpretations are hypothesis-generating and based on preclinical or indirect evidence; the direct causal contribution of PHR remains unproven. Therefore, PHR should be interpreted as a marker of physiological state at admission rather than as a mediator of protection.

Importantly, no established physiological reference range exists for PHR in healthy individuals or patients with intracerebral hemorrhage. As PHR reflects both platelet and HDL-C dynamics, which are influenced by inflammation, metabolic status, and acute stress, its distribution varies across populations. Accordingly, we analyzed PHR using cohort-specific quartiles, and future studies are warranted to define normative ranges and clinically meaningful cutoffs.

This study has several limitations. First, the absence of hematoma volume and location—the strongest prognostic factors in ICH—represents a critical residual confounder, limiting causal inference. PHR may be a surrogate for smaller hematoma size or more favorable location rather than an independent protective factor. Second, as the analysis was inherently restricted to patients surviving to ICU admission, the observed association may be confounded by survivor bias and reverse causation, where a lower PHR could be a consequence rather than a cause of severe hemorrhage. As our cohort included only patients surviving the first 24 h of ICU care, the findings may not generalize to ultra-early deaths. Sensitivity analyses including these early fatal cases showed weaker associations with in-hospital mortality, suggesting that early deaths may influence effect estimates. Third, the retrospective, single-center design limits generalizability, and PHR was measured only once at admission, which may reflect early clinical deterioration rather than purely baseline risk. Fourth, time from ICH onset to ICU admission was unavailable, which may influence PHR measurements. Fifth, subgroup analyses were limited by sample size within strata. Finally, our findings are most applicable to ICH patients with complete admission laboratory data who survive the initial 24 h of ICU care. Future studies should include these imaging variables to better clarify the association between PHR and short-term mortality.

## Conclusion

This study demonstrates an independent, inverse association between PHR and short-term mortality in a large cohort of critically ill ICH patients. Our findings suggest that PHR may serve as a composite biomarker reflecting the balance between hemostatic capacity and vascular protection in the acute phase of ICH. Future studies are needed to validate this association and elucidate the underlying mechanisms.

## Methods

### Data source and setting

This retrospective cohort study utilised data from the Medical Information Mart for Intensive Care IV database (MIMIC-IV version 3.1). The dataset comprises more than 94,000 intensive-care admission records from Beth Israel Deaconess Medical Centre, Boston, MA, collected between 2008 and 2022. Institutional Review Board authorisation for database access was obtained from the Institutional Review Board of the Massachusetts Institute of Technology (Cambridge, MA; IRB No. 0403000206) and the Beth Israel Deaconess Medical Center Institutional Review Board (Boston, MA; IRB No. 2001-P-001699/14)^26^. Author Qiyin Cai provided permission for database utilisation (certification ID 63343183). As the study employed retrospectively collected, de-identified data, the requirement for informed consent was waived by these Institutional Review Boards. All methods were performed in accordance with relevant guidelines and regulations, including the ethical principles of the Declaration of Helsinki and the data use agreements of the MIMIC-IV database.

### Study population

Patients of ICH were ascertained from the International Classification of Diseases, Ninth and Tenth Revisions using codes ICD-9: 431 and ICD-10: I61.0–I61.9 (Table S1)^27^. Exclusion criteria were: (1) absence of ICU-directed treatment during the index hospitalisation; (2) more than one ICH admission (only the first admission per patient was analysed); (3) age < 18 years or ≥ 90 years; (4) incomplete essential data (platelet count, HDL‑C or survival information); (5) ICU stay shorter than 24 h; (6) PHR values beyond mean ± 3SD, which were treated as outliers.

This study was intentionally restricted to critically ill ICH patients admitted to the ICU to ensure clinical comparability and standardized early laboratory assessment. Patients with ICU stays shorter than 24 h were excluded to reduce heterogeneity related to incomplete baseline evaluation and extreme early clinical trajectories.

As a sensitivity analysis, we repeated the analysis in a broader ICU-treated ICH population by applying only exclusion criteria (1)–(4), without restricting ICU length of stay or excluding extreme PHR values (Fig. S1). The results of this sensitivity analysis are presented in Table S9.

### PHR assessment

PHR was defined as the ratio of platelet count (10^9/L) to HDL-C (mmol/L)^28^. PHR was analyzed as a continuous variable, a Z-scores transformation was applied to meet the normal distribution. Platelet count and HDL-C levels were obtained on the first day of ICU admission.

### Outcome

Short-term mortality, defined as a combination of in-hospital and 30-day mortality from ICU admission, served as our primary outcome measure. For 30-day mortality, follow-up was limited to the index hospitalization period, focusing on deaths occurring during the acute ICU phase. All mortality analyses were conducted using all-cause mortality; competing risks from specific causes of death were not formally separated.

### Covariates

The MIMIC-IV database’s patient data were extracted using structured query language (SQL). Clinical knowledge and previous research served as a reference for the variable selection. The following covariates were included: general characteristics (age, gender, race, weight); vital signs (heart rate, respiratory rate, mean arterial pressure [MAP]); comorbidities (hypertension, diabetes); laboratory parameters (hemoglobin, white blood cell count [WBC], platelet count, glucose, prothrombin time, activated partial thromboplastin time [APTT], HDL-C, and creatinine; and scoring systems (Simplified Acute Physiology Score II [SAPS II], Acute Physiology Score Ⅲ [APS III], and Glasgow Coma Scale). Estimated glomerular filtration rate (eGFR) was calculated using the following equation: eGFR = 175 × creatinine^(− 1.234) × age^(− 0.179) × 0.79 (for female patients)^29^.

### Statistical analysis

To assess potential selection bias introduced by the exclusion criteria, we compared the baseline characteristics of the final included patient cohort with all excluded patients. Additionally, among the excluded patients, we further compared those with ICU stays < 24 h to those with ICU stays ≥ 24 h.

Patient demographics and characteristics were summarized according to PHR quartiles. Continuous variables were presented as mean ± standard deviation (SD) if normally distributed, or as median (interquartile range, IQR) otherwise. Categorical variables were reported as counts and percentages. Differences between groups were assessed using ANOVA or the Kruskal-Wallis test for continuous variables, and the chi-square test for categorical variables. For covariates with a missing data proportion of less than 10%, the incomplete cases were deleted (Table S2).

Univariate and multivariable Cox regression analyses were conducted to assess the association between PHR and short-term mortality (in-hospital and 30-day). Variables with *P* < 0.20 in univariate analysis were included in the multivariable model (Table S3), followed by backward stepwise elimination retaining variables with *P* < 0.05. Multicollinearity was assessed using Variance Inflation Factor (VIF), and all included covariates had VIF < 5, indicating no substantial collinearity (Table S4 ). Clinically relevant variables or those changing the PHR estimate by > 10% were also retained. Hazard ratios (HRs) with 95% confidence intervals (CIs) were reported. *P* values in Cox models were derived as follows: for continuous PHR (per 1-SD increase), *P* values were obtained from the Wald test of the standardized regression coefficient; for quartile analyses, Q1 was set as the reference group and *P* values for Q2–Q4 reflected comparisons with Q1; trend tests were conducted by entering PHR quartiles as an ordinal variable (1–4) into the Cox proportional hazards model; the hazard ratio reflects the risk change per quartile increment, with statistical significance evaluated using the Wald test. The proportional hazards assumption was evaluated using Schoenfeld residuals, with no significant violations detected for PHR in either continuous or quartile form (all *P* > 0.05) (Table S5). Two Cox regression models were fitted: Model 1 (unadjusted) and Model 2 (adjusted for age, gender, heart rate, respiratory rate, MAP, hypertension, diabetes, WBC, glucose, APTT, eGFR, SAPS II, and APS Ⅲ).

The dose-response relationship of PHR with in-hospital and 30-day mortality was assessed using restricted cubic spline (RCS) regression to allow for non-linearity. Kaplan-Meier survival curves were generated by PHR quartile, and subgroup analyses were stratified by age, gender, hypertension, and diabetes to investigate effect modification. Effect modification was further evaluated using likelihood ratio tests contrasting models with and without interaction terms, from which *P* values were obtained. Exploratory piecewise Cox regression was used to assess potential threshold effects, comparing segmented models to a single linear model using likelihood ratio tests. Sensitivity analyses assessed the robustness of the results by repeating the Cox models while including patients with ICU stays < 24 h and outliers.

All statistical analyses were conducted using R version 4.2.2 (R Foundation for Statistical Computing; http://www.R-project.org) and Free Statistics version 2.1. Statistical significance was defined as *P* < 0.05.

## Supplementary Information

Below is the link to the electronic supplementary material.

Supplementary Material 1

Supplementary Material 2

Supplementary Material 3

Supplementary Material 4

## Data Availability

The data that support the findings of this study are available from the corresponding author upon reasonable request.

## References

[1] Parry-Jones, A. R. et al. World Stroke Organization (WSO): Global intracerebral hemorrhage factsheet 2025. *Int. J. Stroke*. **20**, 145–150 (2025). 39629687 10.1177/17474930241307876 PMC11786522

[2] Wang, S. et al. Epidemiology of intracerebral hemorrhage: a systematic review and meta-analysis. *Front. Neurol.* **13**, 915813 (2022). 36188383 10.3389/fneur.2022.915813 PMC9523083

[3] Kazui, S., Naritomi, H., Yamamoto, H., Sawada, T. & Yamaguchi, T. Enlargement of spontaneous intracerebral hemorrhage. Incidence and time course. *Stroke* **27**, 1783–1787 (1996). 8841330 10.1161/01.str.27.10.1783

[4] Rajapathy, S. K., Idris, Z., Kandasamy, R., Hieng, A. W. S. & Abdullah, J. M. Inflammatory biomarkers and their value in predicting survival and outcome among patients with spontaneous intracerebral haemorrhage. *Malays J. Med. Sci. : MJMS*. **24**, 51–65 (2017). 28814933 10.21315/mjms2017.24.3.7 PMC5545618

[5] van der Stoep, M., Korporaal, S. J. A. & Van Eck, M. High-density lipoprotein as a modulator of platelet and coagulation responses. *Cardiovasc. Res.* **103**, 362–371 (2014). 24891399 10.1093/cvr/cvu137

[6] Ali, A. et al. Association between HDL levels and stroke outcomes in the arab population. *Sci. Rep.* **14**, 3071 (2024). 38321149 10.1038/s41598-024-53613-z PMC10847494

[7] Wu, W. et al. Impact of platelet-to-HDL-cholesterol ratio on long-term mortality in coronary artery disease patients with or without type 2 diabetes: insights from a chinese multicenter cohort. *J. Inflamm. Res.* **17**, 2731–2744 (2024). 38737110 10.2147/JIR.S458950 PMC11086646

[8] Mandel, J., Casari, M., Stepanyan, M., Martyanov, A. & Deppermann, C. Beyond hemostasis: platelet innate immune interactions and thromboinflammation. *Int. J. Mol. Sci.* **23**, 3868 (2022). 35409226 10.3390/ijms23073868 PMC8998935

[9] Barter, P. J. et al. Antiinflammatory properties of HDL. *Circ. Res.* **95**, 764–772 (2004). 15486323 10.1161/01.RES.0000146094.59640.13

[10] Nofer, J. R. et al. Suppression of endothelial cell apoptosis by high density lipoproteins (HDL) and HDL-associated lysosphingolipids. *J. Biol. Chem.* **276**, 34480–34485 (2001). 11432865 10.1074/jbc.M103782200

[11] Zhao, J. et al. Association between high-density lipoprotein-related inflammation index and periodontitis: insights from NHANES 2009–2014. *Lipids Health Dis.* **23**, 321 (2024). 39342327 10.1186/s12944-024-02312-9 PMC11439298

[12] Wang, B., Wang, J., Liu, C. & Hu, X. The potential of platelet to high-density lipoprotein cholesterol ratio (PHR) as a novel biomarker for heart failure. *Sci. Rep.* **14**, 23283 (2024). 39375501 10.1038/s41598-024-75453-7 PMC11458566

[13] Luo, H., Li, G., Chen, Y., Shen, Y. & Shen, W. Association of platelet-to-high-density lipoprotein cholesterol ratio and its cumulative exposure with cardiovascular disease risk: a prospective cohort study in chinese population. *Front. Cardiovasc. Med.* **12**, 1580359 (2025). 40416811 10.3389/fcvm.2025.1580359 PMC12098545

[14] Chen, P. et al. Platelet to high density lipoprotein cholesterol ratio is associated with diabetes and prediabetes in NHANES 2005 to 2018. *Sci. Rep.* **14**, 30082 (2024). 39627414 10.1038/s41598-024-81637-y PMC11614884

[15] Jin, X. et al. Association of platelet to high density lipoprotein cholesterol ratio with coronary lesion severity in middle aged and elderly adults. *Sci. Rep.* **15**, 27336 (2025). 40717169 10.1038/s41598-025-12981-w PMC12301471

[16] Huang, Z. et al. Elevated platelet count is associated with decreased mortality from hemorrhagic stroke in hospital: a multi-center retrospective cohort study. *Sci. Rep.* **14**, 3797 (2024). 38360953 10.1038/s41598-024-53956-7 PMC10869352

[17] Robert, J., Osto, E. & von Eckardstein, A. The endothelium is both a target and a barrier of HDL’s protective functions. *Cells* **10**, 1041 (2021). 33924941 10.3390/cells10051041 PMC8146309

[18] Huang, Y., Hou, X., Lv, F. & Gong, Z. Association of the platelets to high density lipoprotein cholesterol ratio and risk of heart disease events in middle-aged and elderly chinese population: a retrospective cohort study utilizing the CHARLS database. *Rev. Cardiovasc. Med.* **26**, 26403 (2025). 40026506 10.31083/RCM26403 PMC11868875

[19] Li, W. & Wu, P. The platelet to high-density lipoprotein cholesterol ratio is associated with thyroid hormone abnormalities based on NHANES 2007 to 2012 data. *Sci. Rep.* **15**, 21373 (2025). 40596193 10.1038/s41598-025-06187-3 PMC12217886

[20] Lillemäe, K. et al. Early thrombocytopenia is associated with an increased risk of mortality in patients with traumatic brain injury treated in the intensive care unit: a Finnish Intensive Care Consortium study. *Acta Neurochir. (Wien)*. **164**, 2731–2740 (2022). 35838800 10.1007/s00701-022-05277-9 PMC9519714

[21] Cui, Y. et al. Platelet-to-neutrophil ratio and efficacy of remote ischemic conditioning in acute ischemic stroke. *PLOS One*. **20**, e0322037 (2025). 40608686 10.1371/journal.pone.0322037 PMC12225825

[22] Luo, H. et al. Transfusion of Resting Platelets Reduces Brain Hemorrhage After Intracerebral Hemorrhage and tPA-Induced Hemorrhage After Cerebral Ischemia. *Front. Neurosci.* **13**, 338 (2019). 31024246 10.3389/fnins.2019.00338 PMC6460946

[23] Xu, C. et al. Platelet-membrane-coated polydopamine nanoparticles for neuroprotection by reducing oxidative stress and repairing damaged vessels in intracerebral hemorrhage. *Adv. Healthc. Mater.* **12**, e2300797 (2023). 37310885 10.1002/adhm.202300797

[24] Birjmohun, R. S. et al. High-density lipoprotein attenuates inflammation and coagulation response on endotoxin challenge in humans. *Arterioscler. Thromb. Vasc Biol.* **27**, 1153–1158 (2007). 17303780 10.1161/ATVBAHA.106.136325

[25] Wan Ahmad, W. N. H. et al. Low serum high density lipoprotein cholesterol concentration is an independent predictor for enhanced inflammation and endothelial activation. *PLOS One*. **10**, e0116867 (2015). 25614985 10.1371/journal.pone.0116867 PMC4304817

[26] Johnson, A. E. W. et al. MIMIC-IV, a freely accessible electronic health record dataset. *Sci. Data*. **10**, 1 (2023). 36596836 10.1038/s41597-022-01899-x PMC9810617

[27] Huang, Y., Li, Z. & Yin, X. Triglyceride-glucose index: a novel evaluation tool for all-cause mortality in critically ill hemorrhagic stroke patients-a retrospective analysis of the MIMIC-IV database. *Cardiovasc. Diabetol.* **23**, 100 (2024). 38500198 10.1186/s12933-024-02193-3 PMC10949583

[28] Jialal, I., Jialal, G. & Adams-Huet, B. The platelet to high density lipoprotein -cholesterol ratio is a valid biomarker of nascent metabolic syndrome. *Diabetes Metab. Res. Rev.* **37**, e3403 (2021). 32886844 10.1002/dmrr.3403

[29] Ma, Y. C. et al. Modified glomerular filtration rate estimating equation for chinese patients with chronic kidney disease. *J. Am. Soc. Nephrol. : JASN*. **17**, 2937–2944 (2006). 16988059 10.1681/ASN.2006040368

