# Supplementary material for: Association of platelet to HDL-C ratio with short-term mortality in critically ill intracerebral hemorrhage patients: a MIMIC-IV analysis
Source: Sci Rep. 2026 Mar 10;16:12829. doi: 10.1038/s41598-026-43526-4 (PMC13096478; doi:10.1038/s41598-026-43526-4)
Supplement: Supplementary file 1 — Supplementary Material 1 [file 41598_2026_43526_MOESM1_ESM.docx]

**Association of platelet to HDL-C ratio with short-term mortality in critically ill intracerebral hemorrhage patients: a MIMIC-IV analysis**

Yongtong He^1*^, Qianshan Zhao^1^, Qiyin Cai^2^

^1^Department of Neurosurgery, Affiliated Jiangmen Traditional Chinese Medicine (TCM) Hospital of Ji'nan University, Jiangmen, China.

^2^Department of Pediatric intensive care units, Jiangmen Maternity and Child health care hospital, Jiangmen, China.

*Corresponding Author: Department of Neurosurgery, Affiliated Jiangmen TCM Hospital of Ji'nan University, No.30, Huayuan East Road, Jiangmen, Guangdong 529000, China. Tel: 86–07503509898. Fax: 86–07503509898. E–mail address: heyongtong2024@126.com.

**Supplement Material**

**Table S1 Distribution of patients and admissions by ICD codes for intracerebral hemorrhage.**

**Table S2 Missing Data Frequency and Percentage in the Study Cohort.**

**Table S3 Univariate associations with short-term mortality in ICH.**

**Table S4 Variance Inflation Factor (VIF) Analysis for Covariates in the Fully Adjusted Cox Regression Model.**

**Table S5 Proportional hazards assumption test based on Schoenfeld residuals for PHR with short-term mortality.**

**Table S6 Baseline clinical characteristics of included vs. excluded patients.**

**Table S7 Baseline Characteristics of Excluded Patients, Stratified by ICU Length of Stay (<24 Hours vs. ≥24 Hours).**

**Table S8 Results of Piecewise Regression Analysis for PHR and in-hospital Mortality.**

**Table S9 Sensitivity analyses of association between PHR and short-term mortality in the multiple cox regression model (included length of ICU stay<24 hours and outliers).**

**Table S1 Distribution of patients and admissions by ICD codes for intracerebral hemorrhage.**

| Icd code | Long_title | patient_count | admission_count |
| --- | --- | --- | --- |
| 431 | Intracerebral hemorrhage | 1777 | 1886 |
| I610 | Nontraumatic intracerebral hemorrhage in hemisphere, subcortical | 243 | 247 |
| I611 | Nontraumatic intracerebral hemorrhage in hemisphere, cortical | 533 | 563 |
| I612 | Nontraumatic intracerebral hemorrhage in hemisphere, unspecified | 101 | 101 |
| I613 | Nontraumatic intracerebral hemorrhage in brain stem | 79 | 81 |
| I614 | Nontraumatic intracerebral hemorrhage in cerebellum | 180 | 187 |
| I615 | Nontraumatic intracerebral hemorrhage, intraventricular | 530 | 541 |
| I616 | Nontraumatic intracerebral hemorrhage, multiple localized | 46 | 46 |
| I618 | Other nontraumatic intracerebral hemorrhage | 613 | 633 |
| I619 | Nontraumatic intracerebral hemorrhage, unspecified | 341 | 348 |
| Total |  | 4443 | 4633 |

Note:

ICD-9 code 431 and ICD-10 codes I610–I619 are included. Patient count refers to unique patients, and admission count refers to total hospital admissions.

**Table S2 Missing Data Frequency and Percentage in the Study Cohort.**

| Variable | Missing Frequency | Missing percentage(%) |
| --- | --- | --- |
| Age | 0 | 0 |
| gender | 0 | 0 |
| Race | 0 | 0 |
| Weight | 1 | 0.114 |
| Heart rate | 1 | 0.114 |
| Respiratory rate | 4 | 0.456 |
| MAP | 1 | 0.114 |
| Hypertension | 0 | 0 |
| Diabetes | 0 | 0 |
| Hemoglobin | 0 | 0 |
| WBC | 0 | 0 |
| PT | 7 | 0.7973 |
| APTT | 9 | 1.025 |
| Glucose | 0 | 0 |
| eGFR | 0 | 0 |
| SAPSII | 0 | 0 |
| ASPIII | 0 | 0 |
| GCS | 0 | 0 |

Note:

PHR: platelet to high-density lipoprotein cholesterol ratio; Q: quartile; MAP: mean arterial pressure; WBC: white blood cell count; PT: prothrombin time; APTT: activated partial thromboplastin time; eGFR: estimated glomerular filtration rate; SAPSII: simplified acute physiology score II; ASPIII: acute physiology score III; GCS: glasgow coma scale.

**Table S3 Univariate associations with short-term mortality in ICH.**

| Variable | HR (95%CI) | *P* value |
| --- | --- | --- |
| Age | 1.02 (1.01–1.04) | 0.001 |
| gender | 0.92 (0.65–1.29) | 0.620 |
| Race | 0.75 (0.53–1.06) | 0.106 |
| Weight | 0.99 (0.98–1.00) | 0.245 |
| Heart rate | 1.01 (1.00–1.02) | 0.043 |
| Respiratory rate | 1.13 (1.07–1.19) | < 0.001 |
| MAP | 0.96 (0.94–0.97) | < 0.001 |
| Hypertension | 0.67 (0.48–0.95) | 0.024 |
| Diabetes | 1.19 (0.83–1.7) | 0.347 |
| Hemoglobin | 0.89 (0.82–0.98) | 0.012 |
| WBC | 1.01 (1.00–1.02) | 0.002 |
| PT | 1.01 (1.01–1.01) | < 0.001 |
| APTT | 1.00 (0.98–1.02) | 0.813 |
| Glucose | 1.06 (1.03–1.09) | < 0.001 |
| eGFR | 0.99 (0.98–0.99) | < 0.001 |
| SAPSII | 1.06 (1.05–1.07) | < 0.001 |
| ASPIII | 1.03 (1.02–1.03) | < 0.001 |
| GCS | 0.92 (0.88–0.97) | 0.002 |

Note:

PHR: platelet to high-density lipoprotein cholesterol ratio; Q: quartile; MAP: mean arterial pressure; WBC: white blood cell count; PT: prothrombin time; APTT: activated partial thromboplastin time; eGFR: estimated glomerular filtration rate; SAPSII: simplified acute physiology score II; ASPIII: acute physiology score III; GCS: glasgow coma scale.

**Table S4 Variance Inflation Factor (VIF) Analysis for Covariates in the Fully Adjusted Cox Regression Model.**

| **Variable** | **VIF** | **Comment** |
| --- | --- | --- |
| Age | 1.687 | Yes |
| gender | 1.318 | No |
| Race | 1.276 | Yes |
| Weight | 1.392 | No |
| Heart rate | 1.390 | Yes |
| Respiratory rate | 1.220 | Yes |
| MAP | 1.327 | Yes |
| Hypertension | 1.147 | No |
| Diabetes | 1.900 | Yes |
| Hemoglobin | 1.441 | No |
| WBC | 1.319 | Yes |
| PT | 1.187 | No |
| APTT | 1.185 | No |
| Glucose | 1.771 | Yes |
| eGFR | 1.339 | Yes |
| SAPSII | 1.662 | Yes |
| ASPIII | 3.124 | Yes |
| GCS | 2.437 | No |

Note:

PHR: platelet to high-density lipoprotein cholesterol ratio; Q: quartile; MAP: mean arterial pressure; WBC: white blood cell count; PT: prothrombin time; APTT: activated partial thromboplastin time; eGFR: estimated glomerular filtration rate; SAPSII: simplified acute physiology score II; ASPIII: acute physiology score III; GCS: glasgow coma scale.

### ****Table S5 Proportional hazards assumption test based on Schoenfeld residuals for PHR with s**hort-term mortality**.****

| **Variable** | **Chi-square (χ²)** | **df** | ***P* value** |
| --- | --- | --- | --- |
| **Mortality of in-hospital** |  |  |  |
| PHR continuous | 3.29 | 1 | 0.07 |
| PHR quartile | 1.14 | 1 | 0.29 |
| **Mortality of 30-day** |  |  |  |
| PHR continuous | 0.26 | 1 | 0.61 |
| PHR quartile | 0.01 | 1 | 0.92 |

Note:

Proportional hazards assumption was assessed using Schoenfeld residuals. For both in-hospital and 30-day mortality, P values for PHR—both continuous and quartile-based—were all >0.05, indicating no violation of the proportional hazards assumption. This confirms the validity of the hazard ratios (HRs) reported in the primary Cox regression analyses.

**Table S6 Baseline clinical characteristics of included vs. excluded patients.**

| **Variables** | **Total (n = 2785)** | **Excluded patients (n = 1907)** | **Incluede patients (n = 878)** | ***P* value** |
| --- | --- | --- | --- | --- |
| **General characteristics** |  |  |  |  |
| Age, (years) | 68.8 ± 15.6 | 68.5 ± 16.3 | 69.6 ± 13.8 | 0.074 |
| gender, (female), n (%) | 1303 (46.8) | 908 (47.6) | 395 (45) | 0.197 |
| Race, (white), n (%) | 1651 (59.3) | 1170 (61.4) | 481 (54.8) | 0.001 |
| Weight, (kg) | 79.1 ± 29.4 | 78.4 ± 32.1 | 80.7 ± 22.4 | 0.053 |
| **Vital signs** |  |  |  |  |
| Heart rate, (beats/min) | 80.0 ± 14.1 | 80.2 ± 14.3 | 79.7 ± 13.5 | 0.46 |
| Respiratory rate, (breaths/min) | 18.7 ± 3.2 | 18.7 ± 3.3 | 18.8 ± 2.9 | 0.327 |
| MAP,(mmHg) | 85.4 ± 10.7 | 84.2 ± 10.7 | 88.1 ± 10.2 | < 0.001 |
| **Commodities** |  |  |  |  |
| Hypertension, n (%) | 1712 (61.5) | 1139 (59.7) | 573 (65.3) | 0.005 |
| Diabetes, n (%) | 684 (24.6) | 421 (22.1) | 263 (30) | < 0.001 |
| **Laboratory parameters** |  |  |  |  |
| Hemoglobin, (g/dl) | 12.4 ± 2.0 | 12.2 ± 2.0 | 12.7 ± 1.9 | < 0.001 |
| WBC, (10^9/L) | 10.3 (8.1, 13.4) | 10.6 (8.2, 13.7) | 10.0 (8.0, 12.8) | < 0.001 |
| Glucose, (mg/dl) | 140.0 ± 43.6 | 142.0 ± 43.6 | 136.1 ± 43.3 | < 0.001 |
| PT, (s) | 13.8 ± 6.7 | 14.1 ± 7.7 | 13.2 ± 3.4 | 0.002 |
| APTT, (s) | 30.1 ± 10.0 | 30.5 ± 11.0 | 29.4 ± 7.5 | 0.009 |
| eGFR, (mL/min/1.73 m^2^) | 89.3 ± 39.2 | 89.7 ± 39.8 | 88.4 ± 37.8 | 0.429 |
| **Scoring system** |  |  |  |  |
| SAPSII, (scores) | 34.0 ± 12.3 | 35.0 ± 12.9 | 31.9 ± 10.6 | < 0.001 |
| ASPIII, (scores) | 43.6 ± 21.4 | 44.8 ± 22.7 | 40.9 ± 18.2 | < 0.001 |
| GCS, (scores) | 11.0 ± 3.9 | 10.9 ± 4.1 | 11.1 ± 3.4 | 0.211 |
| **Outcomes** |  |  |  |  |
| In-hospital mortality | 672 (24.1) | 540 (28.3) | 132 (15) | < 0.001 |
| 30-day mortality | 814 (29.2) | 634 (33.2) | 180 (20.5) | < 0.001 |

Note:

MAP: mean arterial pressure; WBC: white blood cell count; PT, prothrombin time; APTT: activated partial thromboplastin time; eGFR: estimated glomerular filtration rate; SAPSII: simplified acute physiology score II; ASPIII: acute physiology score III; GCS: glasgow coma scale.

**Table S7 Baseline Characteristics of Excluded Patients, Stratified by ICU Length of Stay (<24 Hours vs. ≥24 Hours).**

| **Variables** | **Total (n = 1907)** | **<24h (n = 402)** | **≥24 h (n = 1505)** | ***P* value** |
| --- | --- | --- | --- | --- |
| **General characteristics** |  |  |  |  |
| Age, (years) | 68.5 ± 16.3 | 72.6 ± 15.3 | 67.4 ± 16.4 | < 0.001 |
| gender, (female), n (%) | 908 (47.6) | 206 (51.2) | 702 (46.6) | 0.101 |
| Race, (white), n (%) | 1170 (61.4) | 276 (68.7) | 894 (59.4) | < 0.001 |
| Weight, (kg) | 78.4 ± 32.1 | 74.9 ± 21.0 | 79.3 ± 34.4 | 0.017 |
| **Vital signs** |  |  |  |  |
| Heart rate, (beats/min) | 80.2 ± 14.3 | 77.8 ± 15.3 | 80.8 ± 14.0 | < 0.001 |
| Respiratory rate, (breaths/min) | 18.7 ± 3.3 | 18.3 ± 3.2 | 18.8 ± 3.3 | 0.013 |
| MAP,(mmHg) | 84.2 ± 10.7 | 83.5 ± 12.2 | 84.4 ± 10.2 | 0.128 |
| **Commodities** |  |  |  |  |
| Hypertension, n (%) | 1139 (59.7) | 890 (59.1) | 249 (61.9) | 0.309 |
| Diabetes, n (%) | 421 (22.1) | 336 (22.3) | 85 (21.1) | 0.612 |
| **Laboratory parameters** |  |  |  |  |
| Hemoglobin, (g/dl) | 12.2 ± 2.0 | 12.4 ± 1.9 | 12.2 ± 2.0 | 0.043 |
| WBC, (10^9/L) | 10.6 (8.2, 13.7) | 9.8 (7.7, 13.2) | 10.7 (8.3, 13.8) | 0.001 |
| Glucose, (mg/dl) | 142.0 ± 43.6 | 139.2 ± 44.3 | 142.6 ± 43.5 | 0.211 |
| PT, (s) | 14.1 ± 7.7 | 15.1 ± 11.7 | 13.8 ± 6.3 | 0.005 |
| APTT, (s) | 30.5 ± 11.0 | 30.0 ± 11.3 | 30.6 ± 10.9 | 0.342 |
| eGFR, (mL/min/1.73 m^2^) | 89.7 ± 39.8 | 85.3 ± 38.0 | 90.8 ± 40.2 | 0.016 |
| **Scoring system** |  |  |  |  |
| SAPSII, (scores) | 35.0 ± 12.9 | 34.5 ± 12.7 | 35.1 ± 13.0 | 0.406 |
| ASPIII, (scores) | 44.8 ± 22.7 | 37.1 ± 17.9 | 46.9 ± 23.4 | < 0.001 |
| GCS, (scores) | 10.9 ± 4.1 | 13.3 ± 3.0 | 10.3 ± 4.1 | < 0.001 |
| **Outcomes** |  |  |  |  |
| In-hospital mortality | 540 (28.3) | 159 (39.6) | 381 (25.3) | < 0.001 |
| 30-day mortality | 634 (33.2) | 183 (45.5) | 451 (30) | < 0.001 |

Note:

Table S7 summarizes baseline characteristics and mortality outcomes of excluded patients stratified by ICU length of stay, and is presented to evaluate potential selection bias related to the exclusion criteria.

MAP: mean arterial pressure; WBC: white blood cell count; PT, prothrombin time; APTT: activated partial thromboplastin time; eGFR: estimated glomerular filtration rate; SAPSII: simplified acute physiology score II; ASPIII: acute physiology score III; GCS: glasgow coma scale.

**Table S8 Results of Piecewise Regression Analysis for PHR and in-hospital Mortality.**

| PHR | HR (95%CI) | *P* value |
| --- | --- | --- |
| PHR<230 | 0.994 (0.990 – 0.998) | 0.006 |
| PHR>230 | 0.997( 0.988 – 1.007) | 0.589 |
| Likelihood Ratio test | – | 0.028 |

Note:

Hazard ratios (HRs) and 95% confidence interval (CI) were derived from multivariable Cox regression models, adjusted for Model 2 (age, gender, heart rate, respiratory rate, MAP, hypertension, diabetes, WBC, glucose, APTT, eGFR, SAPSII and ASPIII).

HR: hazard ratio; CI: confidence interval; Ref: reference; PHR: platelet to high-density lipoprotein cholesterol ratio; Q: quartile; MAP: mean arterial pressure; WBC: white blood cell count; APTT: activated partial thromboplastin time; eGFR: estimated glomerular filtration rate; SAPSII: simplified acute physiology score II; ASPIII: acute physiology score III.

**Table S9 Sensitivity analyses of association between PHR and short-term mortality in the multiple cox regression model (included length of ICU stay<24 hours and outliers).**

| **Variable** | **Total(n)** | **Model 1** |  | **Model 2** |  |
| --- | --- | --- | --- | --- | --- |
|  |  | **HR (95% CI)** | ***P* value** | **HR (95% CI)** | ***P* value** |
| **Mortality of in-hospital** |  |  |  |  |  |
| Pre 1 SD increase | 999 | 0.84 (0.70–1.02) | 0.076 | 0.77 (0.63–0.94) | 0.010 |
| Quartiles of PHR |  |  |  |  |  |
| Q1 | 250 | 1(Ref) |  | 1(Ref) |  |
| Q2 | 249 | 1.04 (0.69–1.58) | 0.843 | 1.05 (0.68–1.63) | 0.830 |
| Q3 | 250 | 0.69 (0.44–1.09) | 0.112 | 0.65 (0.41–1.05) | 0.080 |
| Q4 | 250 | 0.65 (0.41–1.03) | 0.067 | 0.53 (0.32–0.88) | 0.014 |
| *P* value for trend test |  | 0.85 (0.73–0.98) | 0.022 | 0.79 (0.68–0.92) | 0.003 |
| **Mortality of 30-day** |  |  |  |  |  |
| Pre 1 SD increase | 999 | 0.75 (0.63–0.90) | 0.002 | 0.79 (0.66–0.95) | 0.010 |
| Quartiles of PHR |  |  |  |  |  |
| Q1 | 250 | 1(Ref) |  | 1(Ref) |  |
| Q2 | 249 | 0.71 (0.49–1.02) | 0.067 | 0.97 (0.65–1.43) | 0.865 |
| Q3 | 250 | 0.77 (0.53–1.11) | 0.157 | 0.96 (0.65–1.41) | 0.818 |
| Q4 | 250 | 0.47 (0.31–0.71) | <0.001 | 0.54 (0.34–0.84) | 0.007 |
| *P* value for trend test |  | 0.81 (0.72–0.92) | 0.001 | 0.85 (0.74–0.97) | 0.015 |

Model 1: Unadjusted.

Model 2: adjusted for age, gender, heart rate, respiratory rate, MAP, hypertension, diabetes, WBC, glucose, APTT, eGFR, SAPSII and ASPII.

HR: hazard ratio; CI: confidence interval; Ref: reference; PHR: platelet to high-density lipoprotein cholesterol ratio; Q: quartile; MAP: mean arterial pressure; WBC: white blood cell count; APTT: activated partial thromboplastin time; eGFR: estimated glomerular filtration rate; SAPSII: simplified acute physiology score II; ASPIII: acute physiology score III.
