# Supplementary figures and images for: Association of platelet to HDL-C ratio with short-term mortality in critically ill intracerebral hemorrhage patients: a MIMIC-IV analysis
Source: Sci Rep. 2026 Mar 10;16:12829. doi: 10.1038/s41598-026-43526-4 (PMC13096478; doi:10.1038/s41598-026-43526-4)

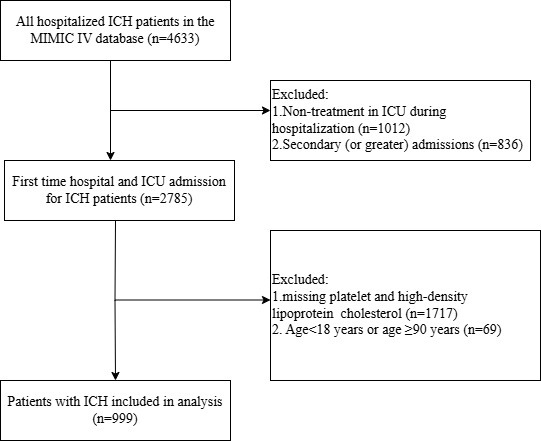

Supplement: Supplementary file 2 — Supplementary Material 2 [file 41598_2026_43526_MOESM2_ESM.jpg]
