# Supplementary material for: Association of platelet to HDL-C ratio with short-term mortality in critically ill intracerebral hemorrhage patients: a MIMIC-IV analysis
Source: Sci Rep. 2026 Mar 10;16:12829. doi: 10.1038/s41598-026-43526-4 (PMC13096478; doi:10.1038/s41598-026-43526-4)
Supplement: Supplementary file 4 — Supplementary Material 4 [file 41598_2026_43526_MOESM4_ESM.docx]

**Fig. S1**. Flowchart of patient selection for the sensitivity

analysis cohort (Table S9).
